# Rare Pediatric Male Presentation of Systemic Lupus Erythematosus With Catastrophic Antiphospholipid Syndrome Following Pulmonary Tuberculosis: A Case Report

**DOI:** 10.1002/ccr3.73362

**Published:** 2026-08-19

**Authors:** Asma Qureshi, Mahaveer Maheshwari, Aasiya Shahbaz Sakarwala, Umaya Memon, Vishan Das, Mumtaz Ali Lakho, Sandesh Kumar, Abdul Ghafoor, Siddhant Kumar Yadav

**Affiliations:** ^1^ Department of Medicine Liaquat University of Medical and Health Sciences Jamshoro Pakistan; ^2^ Liaquat University of Medical and Health Sciences Jamshoro Pakistan; ^3^ Dow University of Health Sciences Karachi Pakistan; ^4^ Patan Academy of Health Sciences Lalitpur Nepal

**Keywords:** anti‐tubercular therapy (ATT), catastrophic antiphospholipid syndrome (CAPS), pulmonary tuberculosis, systemic lupus erythematosus (SLE)

## Abstract

We report a 15‐year‐old with a 3‐year history of polyarthritis, unexplained seizures, and pulmonary tuberculosis who presented with acute neuropsychiatric decline, fever, and thrombocytopenia. Diagnostic workup confirmed systemic lupus erythematosus with triple‐positive antiphospholipid antibodies. Imaging revealed bilateral middle cerebral artery thrombosis, meeting criteria for catastrophic antiphospholipid syndrome triggered by active tuberculosis. Prompt initiation of corticosteroids, anticoagulation, and immunosuppression was critical to achieving clinical stabilization.

## Key Clinical Message

In young patients with unexplained chronic inflammatory symptoms, suspect underlying SLE. Acute neuropsychiatric decline with thrombocytopenia and elevated procalcitonin without infection should prompt urgent evaluation for CAPS, as rapid immunosuppression and anticoagulation are life‐saving.

**Abbreviations**
ANA: antinuclear antibodies
anti‐RNP: anti‐ribonucleoprotein antibodies
anti‐SM: anti‐Smith antibodies
anti‐SSA: Anti‐Sjogren's syndrome‐related antigen A
anti‐β_2_GPI: anti‐β_2_‐glycoprotein I
aPL: antiphospholipid antibodies
APS: Antiphospholipid Syndrome
ATT: anti‐tubercular therapy
CAPS: Catastrophic antiphospholipid syndrome
CT: computed tomography
GCS: Glasgow coma scale
MCA: middle cerebral artery
MRA: magnetic resonance angiography
MRI: magnetic resonance imaging
NF‐κB: nuclear factor kappa B
NSAIDs: nonsteroidal anti‐inflammatory drugs
PTB: pulmonary tuberculosis
SLE: systemic lupus erythematosus
TLR: toll‐like receptor

## 1 Introduction

Systemic lupus erythematosus (SLE) is a multifaceted autoimmune pathology characterized by chronic systemic inflammation and a highly heterogeneous clinical presentation [1]. Within the spectrum of SLE, patients are at risk of developing secondary antiphospholipid syndrome (APS), a rare systemic autoimmune disorder characterized by persistent antiphospholipid antibodies (APL) alongside recurrent arterial or venous thromboembolic events, obstetric complications, and a spectrum of nonthrombotic clinical manifestations [2]. Catastrophic antiphospholipid syndrome (CAPS) constitutes the most acute and fulminant clinical phenotype within the spectrum of APS. It represents a critical escalation of APS's fundamental thromboinflammatory pathology, characterized by the rapid, often simultaneous development of widespread occlusive microthrombosis affecting three or more distinct organ systems over a short temporal window [3].

Based on the molecular and genetic evidence presented, the clinical co‐occurrence of SLE and APS is mechanistically underpinned by a shared pathological triad: a core disruption of immune tolerance leading to pathogenic autoantibody production, consequent activation of the complement system, and the induction of a sustained proinflammatory and prothrombotic endothelial state [4, 5]. This direct pathogenic link has clear clinical correlates; among all reported CAPS cases, approximately one‐quarter (26.9%) occur specifically in patients with underlying SLE, establishing SLE as a major risk factor for this severe thrombotic phenotype [6]. This relationship is notably prominent in younger populations. A systematic review encompassing 386 pediatric patients with APS demonstrated that half had concomitant SLE, with a significant proportion (13%) presenting with the catastrophic variant (CAPS) [7].

The pathogenesis of SLE with CAPS represents a convergence of chronic autoimmunity and an acute thrombotic storm. It originates in the genetically influenced immune dysregulation characteristic of SLE, leading to pathogenic autoantibody production—most critically, anti‐β_2_‐glycoprotein I (anti‐β_2_GPI) antibodies. This results in widespread microvascular thrombosis, neutrophil extracellular trap, and consumptive coagulopathy, culminating in multi‐organ failure. Thus, CAPS in SLE epitomizes the critical intersection of autoimmunity, inflammation, and thrombosis [4, 8].

We present a diagnostically complex case of a 15‐year‐old female admitted with an acute neuropsychiatric crisis and thrombotic events against a background of chronic inflammatory polyarthritis, prior unexplained seizures, and recent pulmonary tuberculosis (PTB). This presentation underscores the interplay between SLE, CAPS, and drug‐induced autoimmunity.

## 2 Case Presentation

### 2.1 Initial Case History/ Examination

We present the case of a 15‐year‐old male patient admitted with an acute, 24‐h history of high‐grade fever, irritability, and generalized tonic‐clonic seizures. The fever was continuous, accompanied by chills and rigors. This acute phase culminated in two witnessed seizures, each lasting approximately 15–30 min, characterized by generalized tonic stiffening followed by flaccidity, eye‐rolling, frothing, and urinary and fecal incontinence. The postictal phase was notable for significant confusion and memory impairment, though no aura, lip smacking, automatisms, or focal neurological deficits were reported. This episode represented the patient's second lifetime seizure event, with a phenomenologically similar first episode occurring 2 years prior, for which he was prescribed levetiracetam 500 mg twice daily.

A detailed review of systems revealed chronic, multi‐system involvement predating the acute neurological crisis, including sicca symptoms (ocular foreign body sensation), photosensitivity, oral ulceration, nonscarring alopecia, transient blurred vision, flank pain, dysuria, and recurrent fungal infections. This constellation of mucocutaneous, renal, ophthalmological, and constitutional findings is critical for establishing an underlying systemic diagnosis.

The patient's past medical history was significant for a 3‐year history of symmetrical inflammatory polyarthritis involving both small and large joints, characterized by prolonged morning stiffness (lasting over 30 min) and joint swelling with tenderness, which paradoxically improved with activity. He had received intermittent corticosteroids and nonsteroidal anti‐inflammatory drugs (NSAIDs) without a formal rheumatological diagnosis. One year before presentation, the patient was diagnosed with PTB, confirmed by chest computed tomography (CT), demonstrating parenchymal involvement with an associated pleural effusion. Rheumatoid factor was not reported. The antitubercular treatment (ATT) included isoniazid at a dosage of 175 mg taken orally every day. However, therapy was prematurely discontinued due to drug‐induced hepatotoxicity (jaundice with deranged liver function tests). Notably, during this period of ATT administration, the patient experienced additional systemic symptoms, including episodes of hematuria, recurrent oral ulceration, and spontaneous epistaxis, which were not fully investigated at the time. This composite history reveals a protracted, evolving multi‐system inflammatory process, with distinct articular, pulmonary, and hematological manifestations, further complicated by a major hepatotoxic reaction to antimicrobial therapy.

**FIGURE 1:**
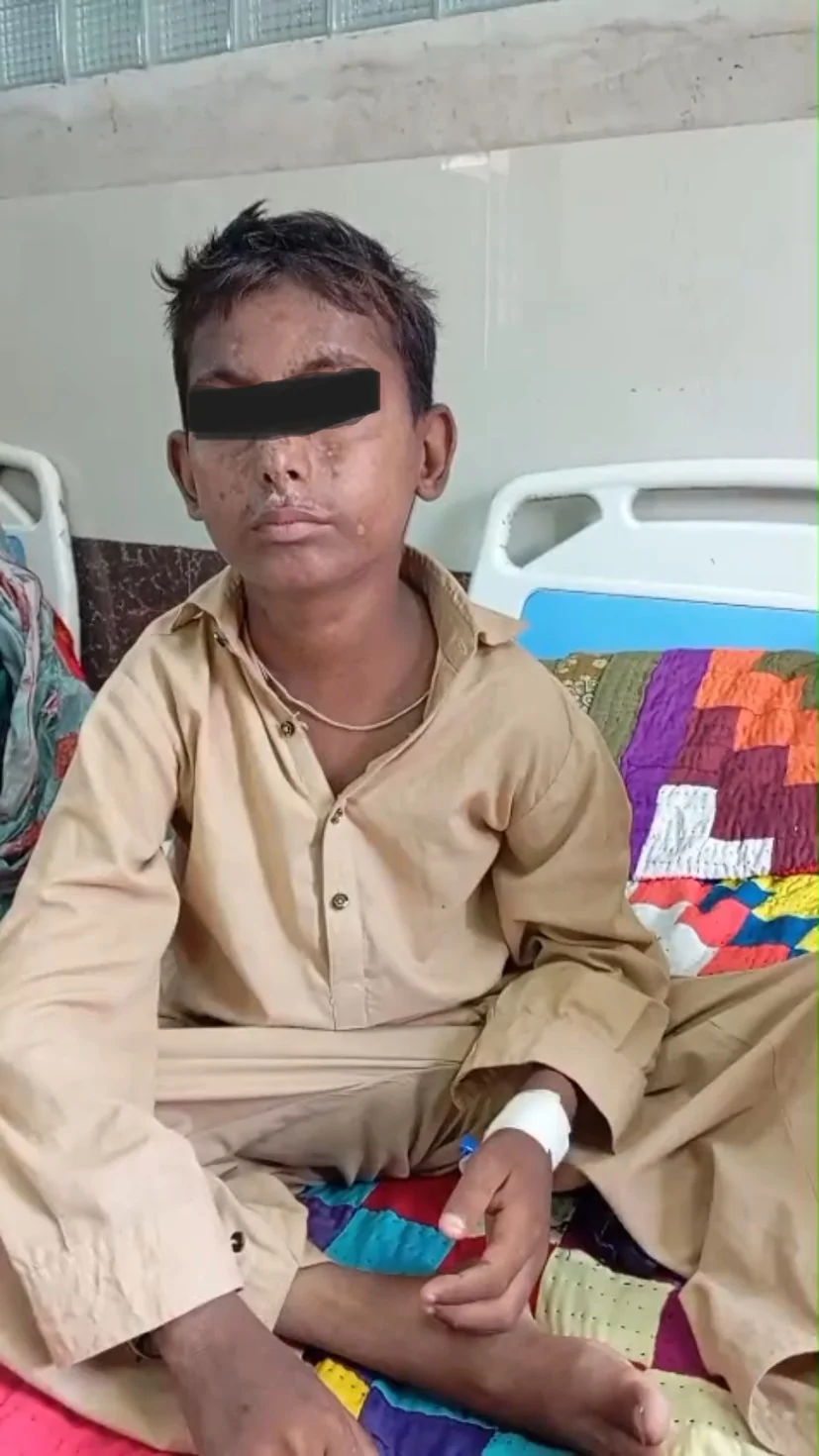
Facial photograph demonstrating a classic malar (butterfly) rash over the cheeks with characteristic sparing of the nasolabial folds, consistent with cutaneous involvement seen in systemic lupus erythematosus.

The patient exhibited prominent mucocutaneous stigmata of systemic autoimmune disease. This included a classic malar (butterfly) rash over the cheeks with sparing of the nasolabial folds, generalized dusky skin discoloration with areas of hyperpigmentation, and patchy nonscarring alopecia on the scalp (Figure 1). The oral cavity revealed multiple aphthous ulcers. Additional findings supportive of chronic disease and potential vasculopathy included onycholysis (separation of the nail plate from the bed) and koilonychia. No lymphadenopathy, thyroid abnormalities, or pedal edema were noted.

The patient's mental status was depressed, with a Glasgow coma scale (GCS) score of 11/15 (E3V3M5). Motor examination showed generalized symmetrical weakness (power 3/5) with normal tone and diminished deep tendon reflexes (1+). The plantar response was mute on the right and flexor (down‐going) on the left. Sensory and cerebellar examinations were intact.

The abdomen was mildly distended and tender on deep palpation. Both kidneys were ballotable. No organomegaly, palpable masses, or hepatic or renal bruits were appreciated. Bowel sounds were present and normoactive.

### 2.2 Laboratory Findings

The patient's laboratory evaluation revealed pancytopenia, including microcytic anemia (hemoglobin 6.6 g/dL, MCV 72.1 fL), leukopenia (3.1 × 10^9^/L), and thrombocytopenia (89 × 10^9^/L). Inflammatory markers were markedly elevated, with ESR 110 mm/h, CRP 23.68 mg/dL, procalcitonin 89.68 ng/mL, and ferritin 165.7 ng/mL. Acute‐phase reactants were markedly deranged with an erythrocyte sedimentation rate of 110 mm/h, C‐reactive protein of 23.68 mg/dL, procalcitonin of 89.68 ng/mL, and ferritin of 165.7 ng/mL. The striking elevation of procalcitonin in the absence of any identifiable infectious source was a critical clue pointing toward the systemic inflammatory response characteristic of CAPS rather than bacterial sepsis.

Serologic testing for autoimmune conditions confirmed SLE with the presence of antinuclear antibodies (ANA), identified through indirect immunofluorescence (IIF), which showed a speckled pattern. The performing laboratory did not provide a report on the ANA titer. The patient also had highly specific anti‐Smith antibodies (anti‐SM++), positive anti‐ribonucleoprotein antibodies (anti‐RNP ++), positive Anti‐Sjogren's syndrome‐related antigen A (anti‐SSA ++), and depressed C4 complement level (0.5 mg/dL). Of note, anti‐double‐stranded deoxyribonucleic acid antibodies (anti‐dsDNA) were negative, a finding observed in a subset of SLE patients without active lupus nephritis. The APL panel demonstrated triple positivity for lupus anticoagulant, anti‐cardiolipin antibodies (IgG/IgM), and anti‐β2 GPI antibodies, establishing the diagnosis of APS and conferring the highest risk for thrombotic complications, including its CAPS.

Renal parameters revealed elevated urea (58 mmol/dL) with preserved creatinine (1.0 mg/dL) and documented hematuria, indicating early renal involvement. The calculated EULAR/ACR score was 28, well above the diagnostic threshold of 10, confirming active SLE with high disease activity.

Collectively, these laboratory findings provided definitive evidence for the dual diagnosis of SLE with lupus cerebritis and CAPS. A complete summary of laboratory results is presented in Table 1.

**TABLE 1:** Summary of laboratory investigations including autoimmune screening markers, antiphospholipid antibody profile, and renal function parameters.

Test name	Result	Normal ranges
**Autoimmune and screening marker**		
Antinuclear antibody (ANA)	Positive	—
Anti‐double‐stranded deoxyribonucleic acid antibody (Anti‐dsDNA)	Negative	—
Anti‐mitochondrial antibody (AMA)	Negative	—
Anti‐smooth muscle antibody (ASMA)	Negative	—
Cytoplasmic anti‐neutrophil cytoplasmic antibody (ANCA‐C PR3)	5.487 RU/mL	Normal < 20
		Elevated > 20
Perinuclear anti‐neutrophil cytoplasmic antibody (ANCA‐P)	4.513 RU/mL	< 20 negative
		> 20 positive
Anti‐cyclic citrullinated peptide antibody (Anti‐CCP)	8.00 U/mL	< 17
**Complement levels**		
C4	05 mg/dL	Female 1–14 years: 13–46 mg/dL
		Female more than 15 years: 15–57 mg/dL
		Male 1–14 years: 14–44 mg/dL
		Male more than 15 years: 15–52 mg/dL
C3	93 mg/dL	Female 1–14 years: 82–173 mg/dL
		Female, more than 15 years: 83–193 mg/dL
		Male 1–14 years: 80–170 mg/dL
		Male more than 15 years: 82–185 mg/dL
**Antiphospholipid antibodies**		
Serum anti‐cardiolipin IgG	55.72 GP/mL	Negative: < 10 GPL‐U/mL
		Equivocal: 10–14.4 GPL‐U/mL
		Positive: > 14.4 GPL‐U/mL
Serum anti‐cardiolipin IgM	81.77 MPL/ml	Negative: < 5 MPL‐U/mL
		Equivocal: 5–7.2 MPL‐U/mL
		Positive: > 7.2 MPL‐U/mL
Serum Beta 2‐Glycoprotein 1 IgM antibody	33.01 U/mL	Negative: < 10 U/mL
		Equivocal: 10–14.4 U/mL
		Positive: 14.4 U/mL
Serum Beta 2‐glycoprotein 1 IgG antibody	22.29 U/mL	Negative: < 10 U/mL
		Equivocal: 10–14.4 U/mL
		Positive: 14.4 U/mL
Lupus anticoagulant (LA) screening	49.9 s	31–44
LA confirmatory	40.8 s	30–38
LA ratio	1.2	0.8–1.1
**Inflammatory marker**		
Procalcitonin	0.251 ng/mL	< 0.05
C‐reactive protein (CRP)	1.43 mg/dL	< 5.0
**Nutritional/metabolic**		
Calcium	7.6 mg/dL	8.1–10.4
Albumin	2.2 g/dL	3.8–4.4
**Enzymes**		
Lactate dehydrogenase (LDH)	356 U/L	230–460
Creatine phosphokinase (CPK)	34 U/L	Female, 24–170
		Male, 24–195
**Renal markers**		
Urea	51 mg/dL	10–50
Creatinine	0.45 mg/dL	0.5–1.3

Abbreviations: IgG, Immunoglobin‐G; IgM, Immunoglobin‐M.

### 2.3 Imaging Findings

**FIGURE 2:**
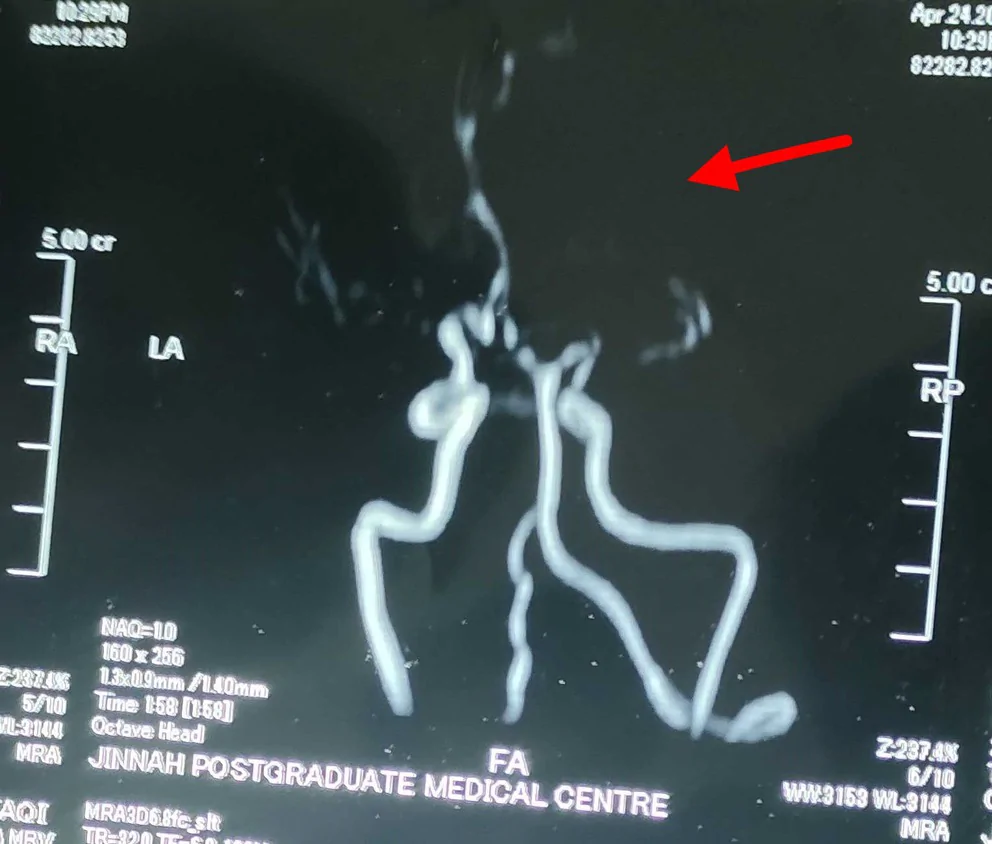
Magnetic resonance angiography (MRA) demonstrating an incomplete circle of Willis with focal signal loss in the left middle cerebral artery (MCA) M1 segment, suggestive of significant stenosis or thrombotic occlusion.

Magnetic resonance imaging (MRI) of the brain revealed ill‐defined T2/FLAIR hyperintense signals in the subcortical and periventricular white matter of the right frontal and parietal lobes, suggesting vasculitis without post‐contrast enhancement. Mild ventricular dilation was noted, with no hemorrhage, mass, or gross infarction. Magnetic resonance angiography (MRA) showed nonvisualization of the distal right middle cerebral artery (MCA) M1 segment, suggestive of thrombus, and focal signal loss in the left MCA M1 segment, indicating stenosis or thrombus (Figure 2). MRV excluded cerebral venous sinus thrombosis. Collectively, these findings confirmed large‐vessel arterial thrombosis involving bilateral middle cerebral artery territories, meeting the CNS criterion for CAPS, while the white matter changes supported underlying lupus cerebritis.

### 2.4 Diagnosis

The autoimmune workup revealed strongly positive ANA with a speckled pattern, along with a positive extractable nuclear antigen (ENA) panel showing anti‐RNP/SM (++), anti‐SM (++), anti‐SSA (++), and anti‐Ro‐52 (+). The presence of anti‐SM antibodies is highly specific for SLE and, together with low C4 complement levels (0.5 mg/dL), confirmed active SLE. Additional supportive findings included normocytic microcytic anemia (hemoglobin 6.6 g/dL), leukopenia (WBC 3.1 × 10^9^/L), thrombocytopenia (platelets 89 × 10^9^/L), elevated ESR (110 mm/h), and disproportionately mild elevation of CRP (23.68 mg/dL), the latter being a classic feature of SLE flares. The EULAR score of 28 further solidified the diagnosis of SLE with lupus cerebritis, explaining the patient's neuropsychiatric manifestations, including seizures and altered mental status.

The diagnosis of CAPS was established based on the triad of thrombocytopenia (platelets 89 × 10^9^/L), multi‐organ involvement evidenced by central nervous system (seizures, GCS 11/15), renal (flank pain, ballotable kidneys, hematuria), and cutaneous (livedo reticularis, dusky skin) manifestations, and the presence of APL, including lupus anticoagulant, anti‐cardiolipin IgG/IgM, and anti‐β2 glycoprotein. The elevated procalcitonin level (89.68 ng/mL), though often associated with bacterial infection, was interpreted in this context as part of the systemic inflammatory response syndrome seen in CAPS, particularly given the absence of a confirmed infectious source. The presence of imaging‐confirmed cerebral artery stenosis provided definitive evidence of arterial thrombosis involving the central nervous system. This, combined with renal and cutaneous involvement, thrombocytopenia, and positive APL, met the diagnostic criteria for CAPS in a patient with underlying SLE.

Ultimately, the integration of clinical features, serological markers, and the EULAR criteria supported the final diagnosis of SLE and CAPS.

### 2.5 Management

The patient was initiated on a combined regimen targeting both the underlying SLE and CAPS. Immunosuppression was achieved with intravenous methylprednisolone 500 mg once daily for 3 days, followed by maintenance therapy with mycophenolate mofetil 500 mg twice daily and hydroxychloroquine (HCQ) 200 mg twice daily. Anticoagulation was administered using enoxaparin 0.4 units/kg subcutaneously once daily to manage the thrombotic complications of CAPS, along with aspirin 75 mg once daily for antiplatelet effect. Empirical broad‐spectrum antibiotic coverage was provided with ceftriaxone 2 g intravenously twice daily and vancomycin 1 g intravenously twice daily to address possible infection as a trigger for CAPS, given the chest imaging findings suggestive of active tuberculosis. Intravenous normal saline was administered for hydration. This dual approach of immunosuppression, anticoagulation, and antimicrobial therapy was tailored to control the autoimmune inflammatory process, prevent further thrombotic events, and cover potential infectious triggers in this critically ill patient.

## 3 Discussion

This report describes a rare and severe presentation of SLE complicated by CAPS in the setting of an active infectious trigger. The coexistence of chronic autoimmune disease with an acute thrombotic storm, particularly in a young male patient, underscores the complexity of diagnosis and management in such scenarios.

In our patient, the presence of long‐standing inflammatory symptoms, including polyarthritis, mucocutaneous involvement, and hematological abnormalities, strongly suggested underlying SLE. The diagnosis was supported by positive anti‐SM antibodies and low complement levels, which are highly specific markers of active disease [9]. Notably, anti‐dsDNA antibodies were negative, which is consistent with previous studies demonstrating that a subset of patients with active SLE may lack this marker, particularly in nonrenal presentations [10]. This finding reinforces the importance of a comprehensive serological profile rather than reliance on a single antibody.

The most critical feature of this case was the rapid progression to CAPS. CAPS is a rare but life‐threatening condition characterized by widespread thrombosis and involvement of multiple organ systems [8]. Based on the International Consensus Criteria, our patient fulfilled criteria for probable CAPS, given the involvement of the central nervous system, renal and cutaneous systems, positive APL, and imaging‐confirmed thrombosis in the absence of histopathological confirmation [11]. Previous studies have demonstrated that triple‐positive APL are associated with a substantially higher risk of thrombosis and adverse outcomes [12]. The coexistence of SLE further increases susceptibility to CAPS through shared mechanisms of immune activation, complement dysregulation, and endothelial injury [8].

The pathophysiology of CAPS is best explained by a two‐hit model. The first hit involves APL that activate endothelial cells and promote a prothrombotic state. The second hit is a precipitating factor such as infection, surgery, or medication exposure [5]. A key aspect of this case is the identification of active tuberculosis as a likely precipitating factor. Registry data suggest that infection is the most common trigger for CAPS, accounting for a substantial proportion of reported cases. In our patient, radiological evidence of PTB, together with markedly elevated inflammatory markers, supports its role as a triggering event. Infection induces endothelial activation, complement amplification, and cytokine release, all of which contribute to the thrombo‐inflammatory cascade observed in CAPS [13]. Additionally, prior drug‐induced hepatotoxicity and immune stress may have further exacerbated the underlying autoimmune condition.

The rapid neurological deterioration observed in this patient is consistent with prior reports describing central nervous system involvement in CAPS, where arterial thrombosis results in acute neurological decline. Neuropsychiatric SLE can present with seizures, altered behavior, and cognitive dysfunction [14]. However, CAPS should be strongly considered when rapid clinical deterioration is accompanied by radiological evidence of vascular occlusion. In this case, MRA demonstrated thrombosis involving bilateral cerebral artery territories, supporting a thrombotic rather than purely inflammatory process. The coexistence of lupus cerebritis and arterial thrombosis illustrates the dual contribution of inflammation and thrombosis to disease pathogenesis.

An additional diagnostic challenge in this case was the markedly elevated procalcitonin level. While procalcitonin is commonly used as a marker of bacterial infection, previous studies have demonstrated that it may also be elevated in severe inflammatory states [15]. This may lead to diagnostic uncertainty and delay appropriate treatment. Therefore, laboratory findings should always be interpreted within the broader clinical and radiological context.

Hematological abnormalities such as pancytopenia are commonly encountered in SLE and APS. Thrombocytopenia is a recognized feature of APS and is paradoxically associated with both bleeding and thrombosis [16]. Anemia and leukopenia reflect immune‐mediated destruction and, in some cases, bone marrow involvement. Together, these findings indicate high disease activity and further support the underlying autoimmune diagnosis.

Management of CAPS requires urgent and aggressive treatment. Current recommendations advocate a combination of anticoagulation, high‐dose corticosteroids, and immunosuppressive therapy [17]. In this case, intravenous corticosteroids were used to control inflammation, while low‐molecular‐weight heparin was administered to prevent further thrombus formation. Hydroxychloroquine was included for its immunomodulatory and antithrombotic properties [18], and mycophenolate mofetil was used to control underlying SLE activity [19]. This approach is consistent with previously reported management strategies for SLE‐associated CAPS.

The presence of active tuberculosis significantly complicated management. Immunosuppressive therapy can worsen infection, whereas untreated infection can perpetuate the inflammatory and thrombotic cascade [20]. Consequently, careful balancing of antimicrobial therapy and immunosuppression is essential. This case further illustrates the importance of identifying and addressing potential triggers when managing CAPS.

Early recognition of CAPS remains essential because of its high mortality rate. Rapid involvement of multiple organ systems, thrombocytopenia, and positive APL should raise clinical suspicion. Imaging plays a crucial role in confirming vascular involvement and guiding management decisions. A multidisciplinary approach is necessary to address the complex interplay between autoimmunity, thrombosis, and infection.

This report has certain limitations. As a single‐patient observation, its findings may not be generalizable. Furthermore, the overlap between SLE, CAPS, and infection makes it difficult to determine the precise contribution of each factor to disease progression. Nevertheless, this report provides valuable insight into the diagnostic and therapeutic challenges associated with this uncommon presentation.

## 4 Patient's Perspective

Profound distress and apprehension were experienced by the patient at presentation, stemming from uncontrolled seizure, persistent high‐grade fever, and the alarming physical changes observed on his own body. A persistent concern was harbored regarding the availability of adequate medical care, given his upbringing in a rural setting where advanced health facilities were seldom accessible. Following a comprehensive assessment and a clear, empathetic explanation of his condition and treatment plan, a notable sense of reassurance was gradually achieved by him. As clinical improvement progressed and the seizure remained controlled, the initial anxiety was progressively replaced by cautious optimism and diminished fear.

## 5 Take‐Home Message

SLE complicated by CAPS represents a rare but life‐threatening pediatric emergency that necessitates prompt recognition and aggressive multidisciplinary intervention to prevent progressive multi‐organ failure and death. Early integration of clinical, serological, and neuroimaging findings is essential for distinguishing this entity from infectious or primary seizure disorders, particularly when atypical features such as treatment‐refractory fever, thrombocytopenia, and unexplained neuropsychiatric decline are present. In settings where access to specialized rheumatology and intensive care may be limited, a high index of suspicion and timely referral to tertiary care centers are critical to avoid diagnostic delays and ensure the initiation of life‐saving immunosuppressive and anticoagulant therapy. This case underscores the importance of considering underlying autoimmunity in any child presenting with unexplained seizures, chronic inflammatory symptoms, or paradoxical laboratory markers such as elevated procalcitonin without identifiable infection, as the delayed recognition has profound neurological and survival implications.

## 6 Conclusion

This case illustrates a rare, severe presentation of SLE with CAPS triggered by active infection. The triad of autoimmunity, thrombosis, and infection complicates a timely diagnosis. Early recognition of multiorgan involvement, thrombocytopenia, and APL is crucial. Prompt initiation of corticosteroids, anticoagulation, and immunosuppression alongside infection management is essential to reduce mortality. An integrated, multidisciplinary approach and heightened clinical awareness are key to improving outcomes.

## Author Contributions

**Vishan Das:** writing – original draft, writing – review and editing, visualization, validation, supervision. **Mumtaz Ali Lakho:** conceptualization, methodology, validation, supervision, resources, writing – review and editing, project administration. **Umaya Memon:** conceptualization, writing – review and editing, writing – original draft. **Siddhant Kumar Yadav:** writing – original draft, writing – review and editing, methodology, project administration. **Mahaveer Maheshwari:** conceptualization, methodology, visualization, writing – review and editing. **Abdul Ghafoor:** writing – review and editing, writing – original draft, conceptualization, resources, supervision. **Asma Qureshi:** conceptualization, validation, writing – review and editing, writing – original draft, supervision. **Aasiya Shahbaz Sakarwala:** conceptualization, investigation, validation, writing – review and editing. **Sandesh Kumar:** writing – original draft, writing – review and editing, validation, methodology, supervision.

## Funding

The authors have nothing to report.

## Ethics Statement

The authors have nothing to report.

## Consent

A written informed consent was obtained from the patient.

## Conflicts of Interest

The authors declare no conflicts of interest.

## Data Availability

Data sharing not applicable to this article as no datasets were generated or analyzed during the current study.
